# Spatial Perception and Navigation in the Absence of Vision

**DOI:** 10.3390/s25030960

**Published:** 2025-02-05

**Authors:** Daniel-Robert Chebat, Maurice Ptito

**Affiliations:** 1 Visual and Cognitive Neuroscience Laboratory (VCN Lab), The Department of Psychology, Ariel University, Ariel 40700, Israel; 2 Department of Neurology and Neurosurgery, Ecole d’Optométrie, Université de Montréal, Montreal, QC H3T 1J4, Canada; maurice.ptito@umontreal.ca; 3 Montreal Neurological Institute, McGill University, Montreal, QC H3A 2B4, Canada; 4 Department of Applied Mathematics and Computer ScienceVisual Computing, Danish Technical University, 2800 Kongens Lyngby, Denmark

In congenital blindness (CB), tactile and auditory information can be reinterpreted by the brain to compensate for visual information through brain plasticity mechanisms triggered by training. Visual deprivation may not cause a cognitive spatial deficit since blind people can acquire spatial knowledge about the environment. This spatial competence takes longer to achieve but is eventually reached through training-induced plasticity. Even though complete visual deprivation leads to volumetric reductions in brain structures associated with spatial learning, blind individuals are still able to navigate. However, the neural structures involved in this function are not fully understood. In this Special Issue, leading experts in the field submitted reviews (2) or original research (8) on spatial navigation in the absence of vision, using a variety of approaches to advance our understanding of multisensory spatial knowledge acquisition. Our aim is for these topics to be explored in people who are congenitally blind, late blind, and sighted who use multisensory cues to perceive spatial information, as well as discussing their abilities, strategies, and corresponding mental representations. We therefore organized the chapters into three cohesive themes to offer readers a structured exploration of the multifaceted dimensions of spatial perception and navigation in the absence of vision. These sections allow for a comprehensive exploration, delving into the understanding of spatial navigation, innovative assistive technologies, and the intricacies of multisensory experience and spatial cognition.

**Theme 1: Understanding Spatial Navigation in the Absence of Vision** **.**

*Heled*, *Elul*, *Ptito*, *and Chebat* delve into the intricate landscape of deductive reasoning and working memory skills within individuals living with blindness. These cognitive functions, vital for executive functioning in everyday life, play a pivotal role in shaping the thought processes and actions of individuals, even more so for those who are visually impaired. The study endeavors to shine a light on the interplay between visual experience, deductive reasoning abilities, and working memory skills, offering a nuanced perspective of cognitive function in the absence of sight. Moreover, it challenges preconceived notions about the role of visual experience in cognitive development and underscores the profound interdependence of deductive reasoning and working memory skills within the visually impaired population. This nuanced exploration serves as a testament to the adaptability and resilience of the human mind, shedding light on the incredible potential for cognitive growth and innovation in individuals who navigate the world without the benefit of sight. In their groundbreaking study, *Real and Araujo* explore the impact of network Quality of Service (QoS) on spatial perception through sensory substitution in navigation systems designed for individuals who are blind and visually impaired. Their research underscores the delicate balance between the acquisition of spatial information and temporal constraints in the rapidly evolving landscape of assistive technologies. By conducting tests with participants under varying delay conditions between motor actions and triggered stimuli, the study unveils a trade-off between spatial information acquisition and performance degradation. Moreover, it sheds light on a learning curve in scenarios marked by impaired sensorimotor coupling. In summary, Real and Araujo’s research advances our understanding of how network QoS influences spatial perception through sensory substitution in navigation systems.

**Theme 2: Innovative Assistive Technologies for Spatial Perception** **.**

*Kilian*, *Neugebauer*, *Scherffig*, *and Wahl* introduce a groundbreaking innovation, the “Unfolding Space Glove”, an open-source sensory substitution device. This remarkable device transforms hand gestures into vibratory stimuli, enabling individuals with blindness to explore and navigate their spatial environment haptically. By providing real-time feedback on the relative position and distance of nearby objects through vibrations on the back of the hand, the Unfolding Space Glove empowers blind users in tasks such as object recognition and wayfinding. What makes this device truly revolutionary is its portability, capacity to operate in diverse lighting conditions, its provision of immediate feedback, and its unobtrusive design, rendering it a promising tool for enhancing mobility and spatial awareness among individuals with visual impairments. In the next paper, *Osiński*, *Łukowska*, *Hjelme*, *and Wierzchoń* present the innovative “Colorophone 2.0”, a wearable color sonification device. This remarkable device has the potential to translate spatial color information into real-time stereo soundscapes. The primary objective is to bridge the sensory gap for individuals with visual impairments, providing them with the ability to perceive and interact with color-rich environments through auditory means. The authors delve into the design and development of this sensory substitution device and present a usability evaluation, highlighting its potential to empower those who cannot directly access visual information. The study by *Bleau*, *Paré*, *Djerourou*, *Chebat*, *Kupers*, *and Ptito* focuses on evaluating the EyeCane, an electronic travel aid, for its effectiveness in helping individuals with visual impairments navigate obstacles. Vision loss significantly impacts a person’s ability to move around safely. Various devices have been developed to assist the visually impaired, and the EyeCane is an inexpensive and easy-to-use ETA designed to detect obstacles within a 2 m range. Participants underwent training and testing using the EyeCane in a life-sized obstacle course containing different types of obstacles, such as cubes, door frames, steps, and posts. All subjects quickly learned to use the EyeCane and successfully completed the trials. Early blind participants were the fastest to navigate the obstacle course, followed by late blind and sighted controls. The EyeCane shows promise as a primary mobility aid for blind navigation, but there are challenges related to its ability to reliably detect lower obstacles on the ground.

**Theme 3: Multisensory Experience and Spatial Cognition** **.**

*Afonso-Jaco and Katz’s* review highlights the transformative potential of auditory exploration in individuals who are blind. They investigate the role of auditory information and virtual reality in the development of spatial cognition for blind individuals. The paper underscores the importance of using spatial audio technology and virtual reality as tools to study higher-level cognitive processes related to spatial cognition in the absence of vision. It also touches upon the influence of early visual experience on spatial abilities among the blind, highlighting the complexity of this topic. The authors provide insights into how blind individuals acquire spatial information, create mental representations of their surroundings, and navigate their environment. This research offers valuable contributions to the field of spatial cognition and holds promise for enhancing the spatial abilities of the visually impaired.

In the next paper, *Lahav* immerses us in the world of virtual reality as an orientation aid for individuals who are blind. Their research investigates the impact of virtual environments on the exploration process, cognitive map construction, and orientation tasks, paving the way for immersive and inclusive navigation solutions. The research focused on comparing interactions with different systems, namely BlindAid, Virtual Cane, and real spaces, to assess their influence on the exploration process, the construction of cognitive maps, and the ability to perform orientation tasks. The results demonstrated that participants could effectively explore unfamiliar spaces, construct cognitive maps, and perform orientation tasks when using both virtual systems and real spaces. This paper underscores the potential of virtual reality systems in aiding the spatial orientation of individuals who are blind. It highlights the value of unique user–interface commands, which enhance exploration, cognitive map construction, and orientation performance. Muteness is a prevalent disability with various technological solutions aimed at transforming mute languages into vocal speech. The study by *Holdengreber*, *Yozevitch and Khavkin*, on an Intuitive Cognition-Based Method for Generating Speech Using Hand Gestures, introduces a novel approach that does not require prior sign language knowledge but instead leverages intuitive cognition to generate speech through hand gestures. This study presents a promising alternative for individuals with speech disabilities, offering an intuitive and adaptive speech generation method. Future enhancements could include real-time adaptive filtering and expanded phoneme recognition to improve fluency and accuracy. Finally, the study by *Leah Fostick and Nir Fink* explores how various hearing protection devices (HPDs) impact sound localization, with a focus on three distinct stimuli: spoken words, pink noise, and gunshots. The findings unearth intriguing insights into the complex interplay between sound source, hearing protection, and the listener’s auditory prowess. They highlight the complexity of sound localization and its relationship with hearing protection, offering insights that resonate with both researchers and individuals with hearing impairments.

As Guest Editors, we extend our heartfelt gratitude to each contributor for their dedication to advancing our understanding of spatial perception and navigation in the absence of vision. These insights not only broaden the horizons of scientific knowledge but also hold promise regarding their ability to enrich the lives of individuals with blindness or visual impairments.

